# Effect of Fluidized Bed Drying, Matrix Constituents and Structure on the Viability of Probiotic *Lactobacillus paracasei* ATCC 55544 during Storage at 4 °C, 25 °C and 37 °C

**DOI:** 10.3390/microorganisms10010074

**Published:** 2021-12-30

**Authors:** Devastotra Poddar, Jon Palmer, Shantanu Das, Manju Gaare, Arup Nag, Harjinder Singh

**Affiliations:** 1 Department of Nutrition, Belda College, Vidyasagar University, Paschim Medinipur 721424, West Bengal, India; 2 Riddet Institute, Massey University, Palmerston North 4442, New Zealand; shantanudas85@hotmail.com (S.D.); a.nag@massey.ac.nz (A.N.); h.singh@massey.ac.nz (H.S.); 3 School of Food and Advanced Technology, Massey University, Palmerston North 4442, New Zealand; J.S.Palmer@massey.ac.nz; 4 Department of Dairy Microbiology, GNP College of Dairy Technology, Sardarkrushinagar Dantiwada Agricultural University, Dantiwada 385506, Gujarat, India; manjugdsc@gmail.com

**Keywords:** *Lactobacillus paracasei*, *Lacticaseibacillus paracasei*, fluidized bed drying, probiotics, probiotic viability, probiotic storage study, probiotic encapsulation, storage temperature, Confocal Laser Scanning Microscopy (CLSM), water activity (a_w_), Scanning Electron Microscopy (SEM)

## Abstract

The stabilization of probiotics for application in non-refrigerated food products is a challenging task. In the present study, probiotic *Lactobacillus paracasei* (*Lacticaseibacillus paracasei*) ATCC 55544 cells were immobilized in a dairy matrix comprising of whole milk powder, skim milk powder, or milk protein isolate using fluidized bed drying technology. The samples were taken out at different drying stages, with an apparent water activity (a_w_) of a_w_ 0.5, a_w_ 0.4, and a_w_ 0.3, respectively, and vacuum-packed to maintain the a_w_ and stored at three different temperatures of 4 °C, 25 °C, and 37 °C. The study evaluated the impact of matrix constituents, milk fat, protein, and carbohydrate on the viability of encapsulated probiotic *L . paracasei* ATCC 55544 during storage for 1 month. The whole milk powder matrix provided superior protection to the bacteria. Confocal Laser Scanning Microscopy (CLSM) was used to investigate the structure of the immobilizing matrix and the location of the probiotic *L. paracasei* cells embedded within the matrix. The CLSM study revealed that the probiotic bacterial cells are mostly embedded as clusters beneath the top layer. We hypothesize that the biofilm-like structure, together with the protective whole milk powder matrix, helps to retain the superior viability of probiotic cells during storage at non-refrigerated storage conditions of 25 °C and 37 °C.

## 1. Introduction

Probiotics are live microorganisms that provide a health benefit to the host upon consumption [1]. Immunomodulation is one way by which a probiotic microorganism provides a health benefit to the host [2]. Consumers in post-COVID 19 pandemics are more interested in procuring immune-boosting food products due to commentaries from medical communities and World Health Organization (WHO), which has received widespread media coverage. Delivery of probiotics through non-refrigerated dry food products, for example, infant formula, malted beverage, or snack bars, may bridge this gap (consumer demand). However, the challenge lies in the loss of viability of probiotics during the shelf life of the food products [3,4], as to claim that food is a “probiotic”, there must be proof of delivery of viable strain(s) at an efficacious dose at the end of shelf-life [5]. One may argue that the delivery of probiotics through fermented milk is the method of choice for the widespread application of probiotics. However, probiotic strains have a slow growth rate and are not competitive with the starter culture strains used in fermented milk preparations [6]. Furthermore, they have a poor refrigerated shelf life. A study reported a three-log reduction in the probiotic strain *Lactobacillus johnsonii* LA1 in fermented milk during two weeks of storage at 4 °C [7].

Immobilization of bacteria via drying technology in a protective matrix remains the most common means to stabilize bacteria. The immobilization in a protective matrix creates a micro-environment that maintains the viability of probiotics in harsh external conditions [8]. The immobilization of probiotic bacteria in a matrix capable of preventing viability loss during storage at elevated temperatures is necessary for the incorporation of probiotics in dry food products [9]. Potentially, both freeze-drying and spray-drying can be used to produce concentrated probiotic powders, but both of these technologies use adverse temperature conditions that are injurious or could even be lethal to the bacteria [9,10,11]. Fluidized bed drying, on the other hand, is an energy-efficient drying technique where the bacteria do not attain the temperature of the air as a result of evaporative cooling. It has been widely used for the production of baker’s yeast and wine yeast [12]. However, to date, fluidized bed drying has not been widely used to produce concentrated viable bacteria for shelf stable food applications. Our recent study showed that probiotic *Lactobacillus paracasei* embedded in whole milk powder matrix using fluidized bed drying showed a better viability at 25 °C compared to spray-dried and freeze-dried bacteria in the same matrix [13]. The composition and structure of the matrix within which the probiotic bacteria are contained are important for the stability of the bacteria. Carbohydrates, protein, and fat all play a protective role for bacteria. Carbohydrates, such as lactose, when used in the drying matrix, are known to protect the cells against drying stress and to substitute for the hydrogen-bonded water in the head group of the phospholipid bilayers present in the bacterial cell membrane [14,15,16,17]. In addition, the formation of a glassy (amorphous) state during drying can impart a very high viscosity, which can act as a protective encapsulation for the bacteria, limiting water and oxygen exchange [14,17]. Crystallization of carbohydrates has been observed during storage at high water activity (a_w_) conditions [15,16], indicating an increased molecular mobility [9,13] and leading to increased bacterial death. The presence of proteins in the matrix has been shown to delay the crystallization of carbohydrates, by maintaining the protective glassy state, thereby reducing the rate of diffusion of sugar molecules to form crystals [18,19,20]. Similarly, the presence of fat in the matrix also delays the crystallization of lactose by reducing the water absorbing properties due to the increased surface hydrophobicity [21,22,23]. Therefore, in this study, fluidized bed drying of bacteria in three dairy matrices, i.e., whole milk powder (WMP), skim milk powder (SMP) and milk protein isolate (MPI) was carried out. WMP represents a matrix rich in fat, protein, and lactose, SMP represents a matrix rich in protein and lactose and devoid of fat, while MPI represents a matrix rich in protein, without fat and lactose. The examination of bacterial viability during storage in these matrices with specific compositions was thought to provide a base for further experiments and a better understanding of the importance of matrix constituents.

In our previous studies, we placed immobilized bacterial powders in a controlled storage environment of different water activity conditions by utilizing a saturated salt solution [13]. However, for industries interested in producing fluidized bed-dried probiotic powders immobilized in a protective matrix, it is not practical to utilize the saturated salt solution for maintaining desired water activity levels. Thus, to carry out the industrial simulation, probiotic *Lactobacillus paracasei* (*Lacticaseibacillus paracasei*) ATCC 55544 immobilized in a WMP, SMP, or MPI matrix were taken out at specific intervals during the fluidized bed drying process, with powders with water activity levels of a_w_ 0.3, a_w_ 0.4, and a_w_ 0.5. We know that water activity represents the energy state of water. Therefore, utilizing powders of the same water activity level was thought to provide a fair comparison of the molecular mobility of the immobilized probiotics in the WMP, SMP, MPI matrices, which may be compared with bacterial viability during storage. The powders collected after reaching the desired water activity levels were vacuum packed in aluminum foil pouches. The vacuum packaging of the powders in the aluminum foil provided water vapor barrier protection, thereby maintaining the water activity levels during storage. The powders were further subjected to a variety of storage temperatures (4 °C, 25 °C, and 37 °C). Confocal Laser Scanning Microscopy (CLSM) was used to study the immobilized bacterial location relative to the matrix constituents.

## 2. Materials and Methods

Whole milk powder (WMP), skim milk powder (SMP), and milk protein isolate (MPI) were obtained from Fonterra Co-operative Group Ltd., Auckland, New Zealand.

### 2.1. Bacterial Growth and Cell Harvesting Conditions

*Lactobacillus paracasei* ATCC 55544, renamed *Lacticaseibacillus paracasei* [24] was grown batch-wise in a 5 L Durham bottle in 55 gL^−1^ MRS medium (Merck KGaA, Darmstadt, Germany) at 37 °C. The fermentation took place under microaerophilic conditions. The cells in the stationary phase (18 h after inoculation) were harvested by centrifugation (10 min at 15,000× *g*, in a Sigma 6–16S centrifuge (Sigma Laborzentrifugen GmbH, Osterode am Harz, Germany). The stationary phase cells were used in the current study, based on previous research findings [25,26], indicating that stationary phase cells provided better viability during storage. The harvested cells were washed in buffered peptone water (5 gL^−1^) (Merck), resulting in a cell pellet containing ~3 × 10^11^ CFU/g of bacterial cells.

### 2.2. Enumeration of Viable Bacteria

deMan, Rogosa, and Sharpe (MRS) agar was used to enumerate viable *L. paracasei* ATCC 55544 present in the powder samples that were stored for a period of up to 4 weeks. The samples were homogenized in sterile buffered peptone water (5 g/L Merck, Darmstadt, Germany) for 5 min using a Stomacher 400 Lab Blender (Seward Medical, London, UK). From this homogenate, decimal serial dilutions were made in the same sterile peptone water and used for microbiological analyses. For the determination of viable cells, diluted samples were pour plated on MRS agar plates (Merck) after a 10-fold serial dilution in peptone water. After solidification of the agar, individual bacterial cells were fixed, which allowed them to multiply during incubation and form colonies. The visible colonies developed after 24–48 h incubation; viable cell counts were determined after 48 h of incubation under aerobic conditions at 37 °C. Colonies counted were then multiplied with the dilution factor to obtain total viable cell counts and recorded as colony forming units (CFU) per gram of product. Three batches of the sample powders were made and analyzed for viability.

### 2.3. Fluidized Bed Drying

The harvested cells were mixed manually with WMP, SMP, or MPI for 10 min. The matrix−bacteria mixture was dried in a laboratory fluidized-bed drier (model Uni-Glatt, Glatt GmbH, Binzen, Germany) with dehumidified compressed air at 40 °C. In the case of the fluidized bed drying of bacterial powders, the inlet air temperature is carefully chosen to maintain a delicate balance between the moisture evaporation from the granulated surface and the migration of moisture through the capillaries from the interior of the granulate to the surface, as dried. If the inlet air temperature is too high, a surface crust formation is generally seen which will prevent the moisture removal from deeper layers to the outside. The crust formation delays the drying process with the increased amount of moisture remaining within the crust, leading to increased viability loss during storage. In the fluidized bed drier, air travels upward through the bed of particles with sufficient velocity to provide fluid-like behavior. The freely suspended particles in the air stream are dried by rapid heat exchange and mass transfer [27]. The powders were taken out at various stages during the drying process to achieve powders with water activity (a_w_) levels of a_w_ 0.3, a_w_ 0.4, and a_w_ 0.5.

### 2.4. Water Activity Measurement

Water activity represents the energy level of water in a product. Water activity is defined as the ratio of the vapor pressure of water in a sample to the vapor pressure of pure water at the sample temperature. *L. paracasei* ATCC 55544 embedded in the protective matrix was periodically taken out during drying to have samples with a water activity of a_w_ 0.3, a_w_ 0.4 or a_w_ 0.5 (±0.01). Water activity was measured using Decagon CX-2 Water Activity (a_w_) Instrument (Decagon Devices Inc., Pullman, WA, USA) at 25 °C.

### 2.5. Moisture Content

One gram of the sample was dried at 102 ± 2 °C in a ventilated drying oven. The mass loss was then measured by weighing before and after 3 h drying and cooling in a desiccator [28]. The moisture content was analyzed for the fluidized bed dried powders with water activity levels of a_w_ 0.3, a_w_ 0.4, and a_w_ 0.5.

### 2.6. Packaging and Storage

Powders produced were immediately vacuum-packed in aluminum foil and stored at three different temperatures; 4 °C, 25 °C, and 37 °C. Vacuum packaging in aluminum foil was performed due to its superior water vapor barrier properties [29], which may help in maintaining the water activity of the powders during storage.

### 2.7. Scanning Electron Microscopy

The surface topology of the encapsulated bacterial powder was studied using Scanning Electron Microscopy (SEM). Dry milk powders were sprinkled onto double-sided tape on an aluminum SEM specimen stub, the loose particles were blown off with a hand air “puffer”, and the samples were sputter-coated with gold and viewed in a FEI Quanta 200 Scanning Electron Microscope. Digital images were saved at the required magnifications.

### 2.8. Confocal Laser Scanning Microscopy

In order to study the location of the bacteria within the matrix, Confocal Laser Scanning Microscopy (CLSM) was employed (please find video in Supplementary Materials). The idea was to locate live and dead bacteria within the matrix. A Confocal Laser Scanning Microscope scans a sample sequentially point by point, line by line, or multiple points at once and assembles the pixel information to one image. As a result, optical slices of the specimen are imaged with high contrast and high resolution in x, y, and z planes. The image stacks can be combined to create a 3D (dimensional) view.

Acridine orange (Sigma), propidium iodide (PI) (Sigma), Nile blue (Sigma), and fast green (Sigma) were the strains used in this study. The cells stained with PI were observed not to take up acridine orange. Researchers had previously reported that the dead cells are not stained by acridine orange [30]. PI is not membrane-impermeable and hence is generally excluded from live cells. In the case of a compromised cell membrane, PI binds with the GC (guanine-cytosine) rich region of DNA and causes dead cells to fluoresce [31]. Nile blue is a lipophilic stain that reacts with the milk fat to generate fluorescence [32]. Fast green, in this case, was used to create a contrast from the acridine orange-stained protein with fast green. All the stains were dissolved in commercially available glycerol-based mounting medium Dako (Dako Corporation, Carpinteria, CA, USA) at 1 mg per mL [32]. The use of a mounting medium as a dye carrier prevented the dissolution of the matrix and aided in the visualization of the intact matrix. The laser intensity was 10%, and the images were taken at an increasing depth from the surface with increments of 0.5 µm. The list of the dyes used in the study is shown in Table 1.

## 3. Results

**Figure 1:**
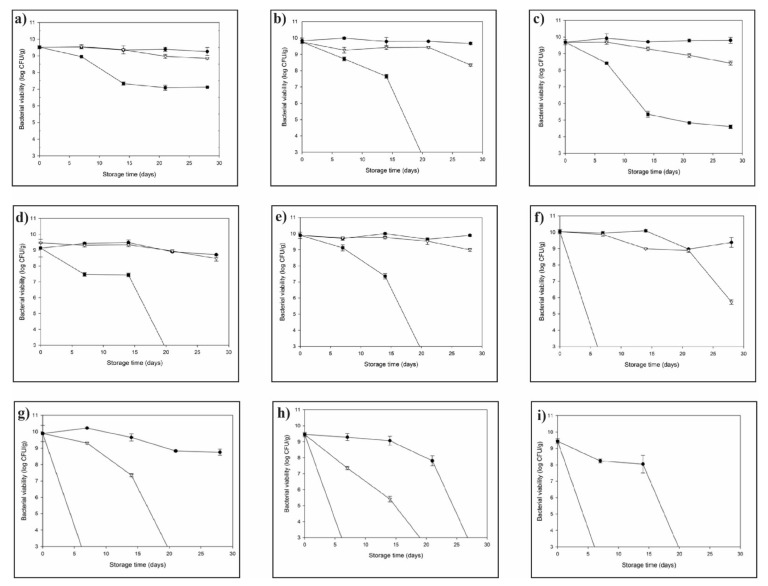
The storage stability of fluidized bed-dried *Lactobacillus paracasei* powders embedded in whole milk powder matrix with (**a**) a_w_ 0.3, (**d**) a_w_ 0.4, (**g**) a_w_ 0.5; skim milk powder matrix with (**b**) a_w_ 0.3, (**e**) a_w_ 0.4, (**h**) a_w_ 0.5; milk protein isolate matrix with (**c**) a_w_ 0.3, (**f**) a_w_ 0.4, (**i**) a_w_ 0.5. The viability is expressed as the logarithmic values of survival against a storage time of 4 weeks at 4 °C (black circle), 25 °C (white triangle), and 37 °C (black square). Error bars represent the standard deviation of means (*n* ≥ 3).

The bacteria embedded in the WMP matrix with a_w_ 0.3 (Figure 1a) had an initial viability count of 9.51 ± 0.09 log CFU/g. During a storage period of 4 weeks, a considerable loss in bacterial viability was observed; the sample stored at 37 °C showed the most considerable loss, where an initial decline to 7.33 ± 0.09 log CFU/g was observed during the 14 days. However, during the subsequent 14-day period, the viability trend remained constant, and the bacteria viability at the end of the storage period was 7.12 ± 0.01 log CFU/g. A mild decline in viability was observed in the samples stored at 25 °C, with 8.95 ± 0.04 log CFU/g of viable bacteria remaining at the end of the storage period. Storage at 4 °C resulted in better bacterial viability (9.28 ± 0.21 log CFU/g at the end of the storage period), as compared to the bacterial samples stored at 25 and 37 °C. 

The bacteria embedded in the SMP matrix with a_w_ 0.3 (Figure 1b) had an initial viability count of 9.75 ± 0.14 log CFU/g. During the storage period of 4 weeks, a considerable loss in bacterial viability was observed; the sample stored at 37 °C showed the most considerable loss where an initial decline till 7.64 ± 0.11 log CFU/g was observed during the first 14-day period. However, an abrupt loss in viability thereafter and less than 3.0 log CFU/g was observed during the subsequent viability check. A mild decline in viability was observed in the samples stored at 25 °C, with 8.34 ± 0.06 log CFU/g of viable bacteria remaining at the end of the storage period. Storage at 4 °C resulted in a better retention of bacterial viability (9.6 ± 0.07 log CFU/g at the end of the storage period), as compared to the bacterial samples stored at higher temperatures of 25 °C and 37 °C, respectively. 

The bacteria in the MPI matrix with a_w_ 0.3 (Figure 1c) had an initial viability count of 9.75 ± 0.15 log CFU/g. During the storage period of 4 weeks, considerable loss in bacterial viability was observed; the samples stored at 37 °C showed the most prominent loss where an initial decline till 5.35 ± 0.18 log CFU/g was observed during the first 14 day period. However, during the next 14 day period, the viability trend remained constant, and the bacterial viability at the end of the storage period was 4.59 ± 0.10 log CFU/g. A slight decline in viability was observed in the samples stored at 25 °C, with 8.24 ± 0.12 log CFU/g of viable bacteria remaining at the end of the storage period. Storage at 4 °C resulted in better bacterial viability (9.68 ± 0.18 log CFU/g at the end of the storage period) as compared to the bacterial samples stored at higher temperatures of 25 and 37 °C. 

The bacteria in the WMP matrix with a_w_ 0.4 (Figure 1d) had an initial viability count of 9.46 ± 0.15 log CFU/g. During the storage period of 4 weeks, considerable loss in bacterial viability was observed; the samples stored at 37 °C showed the most extensive loss where an initial decline till 7.43 ± 0.11 log CFU/g was observed during the first 14 day period. However, during the next 14 day period, there was an abrupt decline and less than 3.0 log CFU/g was observed, till the end of the storage period. The samples stored at 25 °C and 4 °C had a viable count of 8.99 ± 0.16 log CFU/g and 9.12 ± 0.02 log CFU/g, respectively. 

The bacteria in the SMP matrix with a_w_ 0.4 (Figure 1e) had an initial viability count of 9.90 ± 0.19 log CFU/g. In the case of the bacteria stored at 37 °C, a rapid drop in the viable cell count of 7.35 ± 0.17 log CFU/g was observed during the first 14 days period. Thereafter, less than 3.0 log CFU/g was observed till the end of the storage period. The sample stored at 25 °C and 4 °C resulted in better viability results, 8.93 ± 0.08 log CFU/g and 9.89 ± 0.06 log CFU/g, respectively at the end of the storage period. 

The bacteria encapsulated in MPI matrix having a_w_ 0.4 (Figure 1f) had an initial viability count of 9.93 ± 0.12 log CFU/g. Storage at 37 °C resulted in total loss in the bacterial viability within 7 days of storage. The sample stored at 25 °C had a viability count of 5.72 ± 0.14 log CFU/g at the end of the storage period, while the bacteria stored at 4 °C had a viability count of 9.37 ± 0.29 log CFU/g at the end of the storage period.

The bacteria encapsulated in WMP (Figure 1g), SMP (Figure 1h), MPI (Figure 1i) matrix having a_w_ 0.5 had an initial viability count of 9.73 ± 0.09 log CFU/g, 9.44 ± 0.14 log CFU/g and 9.44 ± 0.15 log CFU/g, respectively. Storage at 37 °C resulted in total loss in the bacterial viability within a week of storage. Storage at 4 °C and 25 °C resulted in complete viability loss at the end of the storage period.

**Figure 2:**
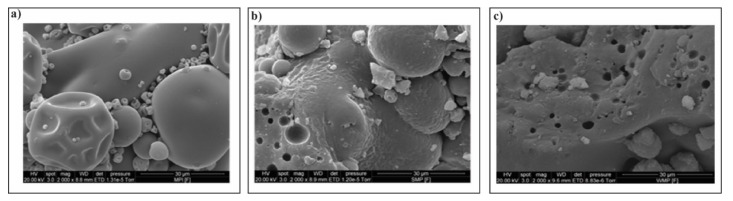
Scanning Electron Microscopy (SEM) images of fluidized bed-dried *Lactobacillus paracasei* powders in MPI (**a**), SMP (**b**) or WMP (**c**).

Scanning Electron Microscopy (SEM) images of the *L. paracasei* embedded in WMP, SMP, and MPI are shown in Figure 2. There were no visible bacteria on the surface of the powders suggesting sufficient embedding of the bacteria within the matrix. The MPI matrix (Figure 2a) had a shiny, lustrous texture and a less rigid structure as compared to the SMP matrix (Figure 2b) and WMP (Figure 2c) matrix. 

**Figure 3:**
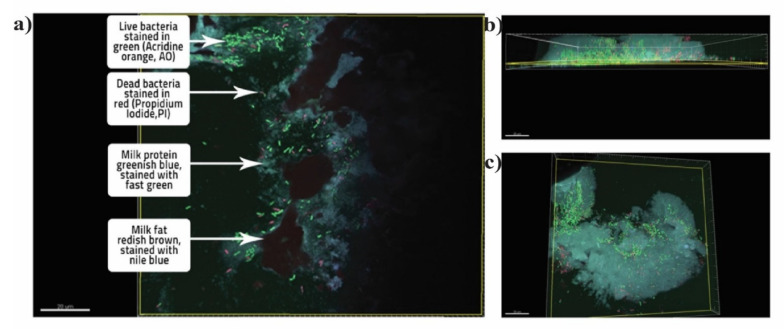
CLSM image of (**a**) surface of the WMP matrix, showing the immobilization of probiotic *Lactobacillus paracasei* within the fat and protein layers (**b**) cross section of the matrix, (**c**) spatial distribution of live and dead bacteria which are located below the surface, comprising of bacteria embedded in the protein and lactose matrix. Green live bacteria, red dead bacteria. The scale bar represents 20 µm.

The matrix structure of the bacteria embedded within the whole milk powder was analyzed using Confocal Laser Scanning Microscopy (CLSM) (Figure 3). The embedded bacteria were observed to lie beneath the surface of the matrix, covered with milk fat. The milk fat could be observed on the surface stained with Nile blue. Milk proteins stained with fast green could be observed to contain the probiotic bacterial mass. Live bacteria were stained with acridine orange, and dead bacteria were stained with propidium iodide and could be observed in red.

## 4. Discussion

The results demonstrate that a higher storage temperature and water activity of the powders leads to increased bacterial death, irrespective of the drying matrices used. The bacterial powders, stored at 4 °C and 25 °C with a_w_ 0.3, and a_w_ 0.4 had greater bacterial viability compared to the powders with a_w_ 0.5 and when stored at 37 °C. This finding is consistent with previously published reports, where the temperature during storage resulted in lower survival rates of spray and freeze-dried *L. paracasei* and *Bifidobacterium* sp. [29,34]. The loss in viability for *Bifidobacterium* spp. was lower upon storage at 4 °C than upon storage at 25 °C for 90 days [35]. Additionally, researchers have observed that increased survival at lower temperatures was possibly due to the lower rate of membrane lipid oxidation, thereby preventing cell membrane damage during storage [36]. A change in the ratio of linoleic/palmitic acid (C18:2/C16:0) or linolenic/palmitic acid (C 18:3/C16:0) has also been linked to the viability loss in freeze-dried bacteria [37]. The formation of lipid hydroperoxides during the storage of bacteria at higher temperatures was also associated with bacterial death upon storage [38].

The a_w_ of the stored powders was necessary for maintaining bacterial viability during storage. With an increase in powder a_w_, an increased rate of bacterial death occurred, and this is consistent with previous findings [36,39]. Powders were vacuum packaged and stored in aluminum foil pouches, based on earlier reports that the vacuum packaging of probiotic powders in aluminum foil laminates, with high water vapor and oxygen barrier properties, improved the bacterial viability during storage [29]. The matrix constituents were found to play an essential role in the survival of *L. paracasei* during storage. The protective carbohydrates present in the probiotic carrier, in an amorphous glassy state, play an essential role by limiting the membrane lipid oxidation, protein unfolding, and chemical reaction, by providing an effective environmental barrier and thereby minimizing the transitional molecular motion [14,17]. 

MPI mainly consists of milk proteins (90%) and is deficient in lactose and fat (3%). The loss of viability was observed in MPI powders with a_w_ 0.3_,_ from 9.75 ± 0.15 to 8.24 ± 0.12 log CFU/g and a_w_ 0.4_,_ from 9.93 ± 0.12 to 5.72 ± 0.14 log CFU/g upon storage at 25 °C. The viability loss in these powders may be attributed to the absence of protective carbohydrate (lactose) in the matrix, which protects the bacteria during drying or desiccation/drying stress. Therefore, the increased bacterial death in the MPI matrix during storage, compared to other matrices, may be due to the more significant cell injury during the drying process (osmotic stress) in the absence of a protective carbohydrate. This may result in an increased number of bacteria dying off during the storage. The MPI matrix has been observed as an outlier in the study carried out by Nag et al., 2019 [40], and the results are in line with our findings.

SMP possess high amount of lactose 54.5%, protein 32.9%, and fat 0.9%, while WMP contains lactose 39.1%, protein 25%, and fat 26.8%. In bacteria embedded in the SMP and WMP matrices, the presence of high amount of lactose would protect the bacterial cells during the drying process by forming an amorphous glass. The amorphous glassy state of carbohydrate imparts a very high viscosity at glass transition temperatures and thereby restricts the molecular mobility in the matrix. Lactose is effective in maintaining the structural and functional integrity of model membranes (microsomes) at low a_w_ (glassy state). The combination of a_w_ 0.3 and temperature of 38 °C is considered as a border point, as below this water activity and temperature combination, lactose remains in the glassy state [41].

Moreover, the presence of proteins in the matrix would also help retain the glassy form of lactose in the SMP and WMP matrices. The presence of fat in the WMP matrix may offer a hydrophobic barrier, thereby helping in maintaining the protective glassy state. It has previously been observed that an encapsulation material containing fat improves the viability of bacteria at high water activities [42]. The WMP matrix provided better protection during storage at an elevated storage temperature than the SMP and MPI matrices where no viable bacterial count could be observed after 4 weeks of storage. The possible reason for the better protective effect of the Whole Milk powder matrix is the lower moisture content (5.19 ± 0.07%) at 0.3 water activity level compared to the SMP (7.26 ± 0.04%) and MPI (7.96 ± 0.05%) matrices (Table 2). The hydrophobicity of fat may have played a role in the lower moisture level in *L. paracasei* immobilized in the WMP matrix.

Moreover, our results are comparable with those obtained by Nag et al., 2019 [40], where the storage of *Lactobacillus reuteri* cells immobilized in the dairy matrix had a viability of 6 log CFU/g at the end of the storage period at 37 °C. The Confocal Laser Scanning Microscopy images indicated that bacteria were present in clusters and just below the top layer. This biofilm-like structure and the protective effect may possibly be enhanced by the presence of exopolysaccharides and the adhesive properties of *L. paracasei* ATCC 55544 [43,44].

## 5. Conclusions

In the present study, a comparison of the fluidized bed drying of probiotic *L. paracasei* in three matrices and upon storage at three temperatures and water activity conditions was carried out. The whole milk powder (WMP) matrix may have provided better protection to the immobilized *L. paracasei* due to the presence of appropriate proportions of fat, protein, and lactose. Our study is of industrial importance. It will offer a guide to an operator carrying out the fluidized bed drying of probiotics regarding the desired water activity levels to be checked during the drying process. Following this study, operators may keep the water activity below a_w_ 0.3 to achieve enhanced viability of the immobilized probiotics. Utilizing Confocal Laser Scanning Microscopy this study also showed how the probiotic *L. paracasei* was immobilized in the WMP matrix by fluidized bed drying. The process of crystallization of a glassy carbohydrate matrix and increased molecular mobility is a simultaneous phenomenon. The crystallization of encapsulating carbohydrates and an increased loss of viability of probiotic bacteria has been observed in many studies. Further investigation is required to explain if a combination of a glassy matrix together with the location of the immobilized bacteria in clusters as observed in this study plays a decisive role in maintaining viability in the case of fluidized bed-dried probiotics. 

## Figures and Tables

**Table 1:** List of Dyes and Excitation–Emission Filters used in this Study.

Dye	Excitation/Bandpass Filter	Function	Reference
Acridine Orange	488/500–540	To stain live, recoverable, growth responsive, metabolically active, dormant and active cells	[30]
Propidium iodide	488/550–620	To stain dead cells	[31]
Nile blue	488/550–620	To stain fat	[32]
Fast Green	633/650–700	To stain protein	[33]

**Table 2:** Water Activity and Product Moisture Content of Powders Obtained with Fluidized Bed Drying Using Different Encapsulating Matrices.

SL/No.	Matrix	Water Activity (a_w_)	Moisture (%)
1	MPI	0.303 ± 0.010	7.96 ± 0.05
2	MPI	0.403 ± 0.010	8.82 ± 0.08
3	MPI	0.510 ± 0.001	11.60 ± 0.12
4	SMP	0.291 ± 0.002	7.26 ± 0.04
5	SMP	0.395 ± 0.004	8.52 ± 0.08
6	SMP	0.495 ± 0.005	12.73 ± 0.13
7	WMP	0.296 ± 0.002	5.19 ± 0.07
8	WMP	0.396 ± 0.009	6.22 ± 0.27
9	WMP	0.487 ± 0.001	10.41 ± 0.14

## Data Availability

Confocal Laser Scanning Microscopy (CLSM) video of sections found at Supplementary Materials.

## Supplementary Materials

The following are available online at https://www.mdpi.com/article/10.3390/microorganisms10010074/s1. Confocal Laser Scanning Microscopy (CLSM) video of sections has been uploaded as Supplementary Files.
